# Academic Engagement of Women as Orthopaedic Surgeons at the Annual Meetings of the Japanese Orthopaedic Association From 2012 to 2022

**DOI:** 10.7759/cureus.57474

**Published:** 2024-04-02

**Authors:** Satomi Nagamine, Tadatsugu Morimoto, Yumi Niizeki, Kazuyo Yamauchi, Naomi Oizumi, Hirohito Hirata, Shiori Tanaka, Masatsugu Tsukamoto, Takaomi Kobayashi, Masaaki Mawatari

**Affiliations:** 1 Department of Orthopaedic Surgery, Saga University, Saga, JPN; 2 Department of Orthopaedic Surgery, Soka Municipal Hospital, Soka, JPN; 3 Department of Community-Oriented Medical Education, Graduate School of Medicine, Chiba University, Chiba, JPN; 4 Upper Extremity Center of Joint Replacement and Endoscopic Surgery, Hokushin Orthopaedic Hospital, Sapporo, JPN

**Keywords:** quota system, orthopaedics, negative stereotype, japanese orthopaedic association, gender diversity

## Abstract

Background:Higher gender diversity correlates with higher patient satisfaction, higher-quality medical education, increased research productivity, and higher revenues. Although the field of Japanese orthopaedic surgery includes the lowest proportion of women and lags in gender diversity, reports on the current gender diversity status in academic activities are scarce. We investigated changes in women's participation in academic activities at the Japanese Orthopaedic Association (JOA) annual meetings over the past 11 years.

Methods:Data on the percentage of women in the JOA membership during 2012-2022 were analyzed to ascertain the proportion of women as first authors of oral and poster presentations, abstract reviewers, invited lecturers, seminar lecturers, general abstract oral chairpersons, symposium chairpersons, and speakers. Regarding the ratio of women among the JOA members during 2012-2022, we relied on data provided by the JOA. Data related to other categories were collected from the abstract book presented at the JOA Annual Meetings between 2012 and 2022. We analyzed the time trend for women's proportions using the Cochran-Armitage trend test. A p-value < 0.05 was considered statistically significant.

Results:During 2012-2022, the percentage of female members (4.9-6.7%), poster first authors (2.7-4.3%), abstract reviewers (0-1.5%), general abstract oral chairpersons (0-2.3%), symposium chairpersons (0-3.6%), and symposium speakers (1.6-6.8%) had increased significantly (p < 0.05). Oral first authors (2.2-4.1%), invited lecturers (0-6.8%), or seminar lecturers (0%-6.7%) showed no trend. Women engaged in academic activities at all annual association meetings did not exceed the women's proportion among the association members.

Conclusion:Although the proportion of women members of the JOA has gradually increased and more women are involved in its annual meetings, the proportion of female presenters, invited speakers, symposiasts, and chairpersons of oral and poster presentations is generally lower than that of women as JOA members. Members should be asked to raise awareness, including more active education of women as physicians in educational institutions and the creation of positive actions to select women as physicians for more important roles (chairpersons, educational speakers, and symposiasts) in the organization of annual meetings.

## Introduction

Diversity is the understanding and acceptance of differences in race, nationality, gender, religion, and values. It indicates a state by which people with various attributes coexist in an organization or group. Diversity can be categorized into superficial diversity, such as nationality, gender, and age, which are easily judged from the outside, and in-depth diversity, such as values, abilities, and lifestyles, which are difficult to judge from the outside. Diversity management aims to create an organizational culture where diverse human resources can maximize their abilities and be fairly evaluated.

Reflecting the recent increase in the number of women as medical students [1], the percentage of women with medical licenses in Japan in 2020 increased to 22.8%. Moreover, the percentage of women with medical licenses under 30 years of age in particular reached 36.2%; these figures are likely to increase in the future [2]. However, the surgical departments in Japan do not reflect this growth rate in women with medical licenses. Although women may or may not be suitable for this field depending on the nature of the specialized field and the characteristics of the department [3], higher gender diversity of physicians has generally been found to correlate with higher patient satisfaction, higher-quality medical education, increased research productivity, and higher revenue [4,5]. The Japanese Orthopaedic Association (JOA) launched the Gender Equality Committee in 2018 to promote the development of orthopaedics by utilizing a diverse workforce regardless of gender. The Gender Equality Committee has conducted surveys and made proposals regarding the working styles of its members, providing information on life events and work-life balance, introducing the appeal and potential of orthopaedic surgery to medical students and residents, promoting career support, and undertaking many other initiatives [2]. Although the percentage of women as orthopaedic surgeons in Japan increased from 4.1% in 2010 to 5.7% in 2020, this remained the lowest percentage of women as physicians among all departments during the study period [6]. Orthopaedics is the medical department with the largest gender gap in Japan.

The age demographics of the male orthopaedic population in 2020 showed a large number of physicians in their 50s, followed by fewer physicians in their 40s and 30s, reflecting a decreased number of young male orthopaedic surgeons [6]. If the number of new young male orthopaedic surgeons continues to decline, there could be a shortage of orthopaedic surgeons in the future. Given the increasing number of women as medical students [1,2], training more women as orthopaedic surgeons is urgently needed. However, among all surgical departments in Japan, orthopaedic surgery is expected to take the longest, 160 years, to achieve the 30% gender diversity goal at this annual rate of change [6], which is far from desirable.

To increase gender diversity in orthopaedic surgery in other countries, annual changes in the role of women in academic activities have been investigated and clarified [5,7-13]. Regarding the percentage of women as physicians in major JOA-related societies in 2020, the top three were the Japanese Pediatric Orthopaedic Association (15%), the Japanese Society for Surgery of the Hand (12%), and the Japanese Society for Surgery of the Foot (8%). The bottom three were the Japanese Hip Society (4.5%), the Japanese Society for Joint Diseases (4.3%), and the Japanese Society for Spine Surgery and Related Research (2.1%) [4]. Thus, gender diversity varies by subspecialty among major JOA-related societies. Although approximately 30% of women (orthopaedic surgeons) in Japan are interested in academic activities, they are unable to fully engage in them due to childbirth and childcare; the details are not fully understood [14]. Despite these issues, only a few reports on orthopaedic surgery gender diversity in Japan have been published in the English literature.

Hence, the initial measure should be carrying out a survey to enhance the understanding and recognition of the matter of gender diversity in the field of orthopaedics in Japan. This study aimed to assess changes in women's involvement in academic activities at the JOA annual meetings from 2012 to 2022.

## Materials and methods

Study design

We performed a retrospective analysis of the data collected from the JOA and publicly accessible data obtained from the abstracts presented at the JOA annual meetings between 2012 and 2022. Since this analysis involved a secondary evaluation of de-identified aggregate data, a formal ethics committee approval was not deemed necessary. Permission was obtained from the JOA for the publication of the data used in this study.

Data extraction

Regarding the ratio of women among JOA members during 2012-2022, we relied on data provided by the JOA. Data related to other categories were obtained from the abstract book presented at the JOA Annual Meetings between 2012 and 2022. 

The inclusion criterion was women as orthopaedic surgeons. We scrutinized the proportion of women who were the first authors of oral and poster presentations, abstract reviewers, invited lecturers, seminar lecturers, general abstract oral chairpersons, symposium chairpersons, and speakers. Furthermore, to investigate which fields involved more women, the subspecialty of the first author for oral and poster presentations (i.e., spine, trauma, sports, rheumatoid arthritis, paediatrics, foot and ankle, hand and elbow, shoulder, hip, knee, osteoporosis, basic research, tumour, rehabilitation, pain, and others including education and locomotive syndrome) was investigated. Subspecialty classification was determined based on the abstract book, with some reference to the review by Pechlivanidou et al. [3]. When abstracts overlapped in subspecialties, classification was based on session categorization in the abstract book.

The exclusion criteria were women who were not orthopaedic surgeons (e.g., pharmaceutical company researchers, basic researchers, and sports doctors other than orthopaedic surgeons), presenters from overseas, and men.

The classification of the subspecialty was based on sessions of the abstract book. We determined the gender from the names of the speakers. However, in cases wherein it was difficult to determine from the names, a manual search was conducted using a search engine (e.g., Google (Alphabet Inc., Mountain View, California, United States)). This manual search was conducted by two independent reviewers.

Statistical analyses

We analyzed the time trend of women's percentage using the Cochran-Armitage trend test from 2012 to 2022. A positive statistical test result (Z > 0) indicated an increasing trend, whereas a negative test result (Z < 0) indicated a decreasing trend. Descriptive statistics were used to analyze subspecialty data. A p-value of 0.05 was considered statistically significant. Data analysis was performed using JMP Pro version 16.2.0 (570548) software (SAS Institute, Cary, North Carolina, United States). Three researchers (SN, TM, and TK) analyzed the data.

## Results

Women's ratio trends from 2012 to 2022

**Figure 1:**
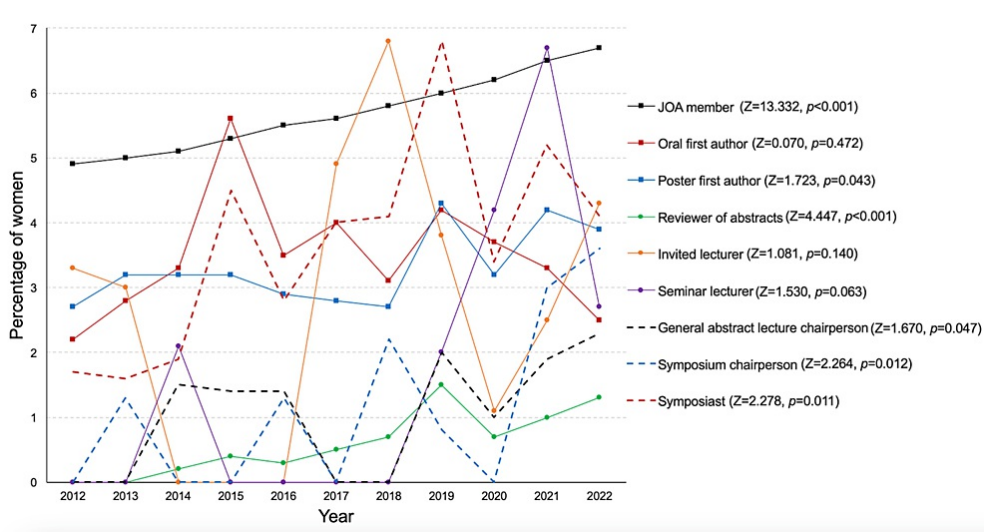
Time trends of the ratio of women in the JOA from 2012 to 2022. JOA, Japanese Orthopaedic Association

Figure 1 illustrates the time trend in the ratio of women in the JOA meetings from 2012 to 2022. The details are presented in Table 1. The ratio of women among JOA members gradually increased from 4.9% to 6.7% (Z = 13.332, p < 0.001) at a rate of increase of approximately 2% per decade. Similarly, between 2012 and 2022, increasing trends were observed in poster first authors (range: 2.7-4.3%) (Z = 1.723, p = 0.043), abstract reviewers (range: 0-1.5%) (Z = 4.447, p < 0.001), general abstract oral chairpersons (range: 0-2.3%) (Z = 1.670, p = 0.047), symposium chairpersons (range: 0-3.6%) (Z = 2.264, p = 0.012), and symposium speakers (range: 1.6-6.8%) (Z = 2.278, p = 0.011). Conversely, no significant trend in oral first authors (range: 2.2-4.1%) (Z = 0.070, p = 0.472), invited lecturers (range: 0-6.8%) (Z = 1.081, p = 0.140), and seminar lecturers (range: 0-6.7%) (Z = 1.530, p = 0.063) was observed. During this period, the proportion of women engaged in academic activities at all annual JOA meetings never exceeded the proportion of female JOA members.

**Table 1:** Time trends of the ratio of women in the JOA from 2012 to 2022. The time trend of representation of women was determined using the Cochran–Armitage trend test. All values are presented as percentages (number of females/overall number). JOA, Japanese Orthopaedic Association

Year	JOA member	Oral first author	Poster first author	Abstract reviewer	Invited lecturer speaker	Seminar lecturer speaker	General abstract oral chairperson	Symposium chairperson	Symposium speaker
2012	4.9 (1,131/23,190)	2.2 (9/402)	2.7 (15/567)	0 (0/573)	3.3 (2/61)	0 (0/56)	0 (0/76)	0 (0/58)	1.7 (2/121)
2013	5.0 (1,167/23,372)	2.8 (12/426)	3.2 (30/947)	0 (0/579)	3.0 (2/66)	0 (0/64)	0 (0/71)	1.3 (1/76)	1.6 (3/185)
2014	5.1 (1.205/23,703)	3.3 (13/396)	3.2 (30/972)	0.2 (1/585)	0 (0/55)	2.1 (1/48)	1.5 (1/66)	0 (0/60)	1.9 (3/160)
2015	5.3 (1,262/24,015)	5.6 (26/467)	3.2 (32/997)	0.4 (2/520)	0 (0/63)	0 (0/56)	1.4 (1/71)	0 (0/64)	4.5 (7/155)
2016	5.5 (1,331/24,384)	3.5 (15/426)	2.9 (29/1003)	0.3 (2/594)	0 (0/60)	0 (0/55)	1.4 (1/71)	1.3 (1/78)	2.8 (8/285)
2017	5.6 (1,367/24,615)	4.0 (14/354)	2.8 (34/1202)	0.5 (3/636)	4.9 (4/81)	0 (0/40)	0 (0/59)	0 (0/68)	4.0 (7/174)
2018	5.8 (1,456/24,918)	3.1 (23/732)	2.7 (27/985)	0.7 (4/560)	6.8 (6/88)	0 (0/48)	0 (0/111)	2.2 (3/134)	4.1 (13/315)
2019	6.0 (1,513/25,126)	4.1 (27/661)	4.3 (44/1021)	1.5 (9/613)	3.8 (3/79)	2.0 (1/49)	2.0 (2/100)	0.8 (1/123)	6.8 (21/307)
2020	6.2 (1,602/25,659)	3.7 (21/565)	3.2 (34/1100)	0.7 (4/553)	1.1 (1/90)	4.2 (2/48)	1.0 (1/100)	0 (0/141)	3.4 (13/383)
2021	6.5 (1,676/25,836)	3.3 (23/698)	4.2 (35/829)	1.0 (7/700)	2.5 (2/79)	6.7 (4/60)	1.9 (4/212)	3.0 (4/132)	5.2 (17/328)
2022	6.7 (1,756/26,197)	2.5 (13/523)	3.9 (42/1090)	1.3 (9/681)	4.3 (4/94)	2.7 (2/73)	2.3 (4/174)	3.6 (5/140)	4.1 (15/363)
p-value	< 0.001	0.472	0.043	< 0.001	0.140	0.063	0.047	0.012	0.011
Z-score	13.332	0.070	1.723	4.447	1.081	1.530	1.670	2.264	2.278

Number of female JOA annual meeting presenters (i.e., the first author for oral and poster presentations) by subspecialty from 2012 to 2022

The top three subspecialties were rheumatoid arthritis (n=4), spine (n=3), knee (n=3), and others (n=3) in 2012; others (n=6), rheumatoid arthritis (n=5), and paediatrics (n=5) in 2013; spine (n=6), knee (n=6), and paediatrics (n=5) in 2014; hip (n=8), rheumatoid arthritis (n=6), and hand and elbow (n=6) in 2015; hip (n=6), knee (n=6), shoulder (n=5), and others (n=5) in 2016; spine (n=6), knee (n=6), sports (n=5), hip (n=5), and osteoarthritis (n=5) in 2017; rheumatoid arthritis (n=8), paediatrics (n=7), knee (n=6), and others (n=6) in 2018; knee (n=12), spine (n=8), and paediatrics (n=8) in 2019; paediatrics (n=11), knee (n=9), osteoporosis (n=5), and tumour (n=5) in 2020; spine (n=10), knee (n=10), and tumour (n=7) in 2021; and trauma (n=9), spine (n=6), and paediatrics (n=6) in 2022. During this period, knee, paediatrics, and spine were the top three most frequently represented subspecialties among women as participants in the JOA meetings, occurring eight, six, and six times, respectively (Table 2). 

**Table 2:** Number of women as JOA annual meeting presenters (i.e., the first author for oral and poster presentations) by subspecialty. *Top three subspecialties for each year

Year	Spine	Trauma	Sports	Rheumatoid arthritis	Paediatrics	Foot and ankle	Hand and elbow	Shoulder	Hip	Knee	Osteoporosis	Basic research	Tumour	Rehabilitation	Pain	Others
2012	3*	1	1	4*	1	2	1	1	0	3*	0	0	1	1	2	3*
2013	2	0	0	5*	5*	1	4	1	4	4	3	1	3	1	2	6*
2014	6*	1	2	1	5*	0	2	2	2	6*	1	2	3	3	3	4
2015	4	2	5	6*	5	1	6*	5	8*	5	3	1	2	0	2	3
2016	3	1	2	1	4	0	1	5*	6*	6*	3	1	3	1	2	5*
2017	6*	2	5*	4	4	1	2	4	5*	6*	5*	0	1	0	1	2
2018	1	2	3	8*	7*	1	1	1	5	6*	4	0	2	2	1	6*
2019	8*	3	4	3	8*	1	5	5	7	12*	3	1	4	1	2	4
2020	2	4	2	4	11*	1	3	2	4	9*	5*	0	5*	0	2	1
2021	10*	2	1	3	5	1	4	1	5	10*	2	0	7*	2	0	5
2022	6*	9*	3	2	6*	2	5	3	2	4	4	1	4	1	0	3

## Discussion

This study revealed three main findings regarding the role of women in the JOA annual meetings from 2012 to 2022. First, we found an increase in the ratio of women among members of the JOA, including poster first authors, abstract reviewers, general abstract oral chairpersons, symposium chairpersons, and symposium speakers. Second, we found no significant trend in terms of oral first authors, invited lecturer speakers, and seminar lecturer speakers. Third, the knee, paediatrics, and spine were the top three most frequently represented subspecialties. 

The roles of women at the JOA annual meetings can be divided into two categories: ordinary meeting participants (oral and poster first authors) and those requested by the meeting organizers (abstract reviewers, invited lecturer speakers, seminar lecturer speakers, general abstract lecture chairpersons, symposium chairpersons, and symposium speakers).

Ordinary meeting participants

We observed a significant increase in the number of poster first authors over time (Z = 1.723, p = 0.043). However, no significant increase was found in the number of oral first authors (Z = 0.070, p = 0.472). Generally, the percentage of women in these roles had not reached the percentage of women as members of the JOA. The ratio of women as JOA members has also been slowly increasing, despite increasing at a very low rate compared with the growth rate of women as physicians overall in Japan. However, the number of women as first authors of oral presentations accepted by meeting organizers has not increased. Although we observed an increasing trend among poster first authors, the ratio was significantly lower than that of women as JOA members for all the years investigated. There are two possible explanations for this finding. First, a large proportion of women as orthopaedic surgeons are still in their early careers and have not engaged in academic activities compared with male orthopaedic surgeons. Second, there may be a lack of education for women (orthopaedic surgeons) in educational and medical institutions.

In this context, Saka et al. reported a low proportion of women as first and last authors in papers published in the Journal of Orthopaedic Science, the JOA's official English-language journal, and emphasized the issue of a lack of mentorship [15]. Saka et al. also suggested that research mentoring programs or workshops, particularly for women as orthopaedic surgeons, could be one approach for compensating for the lack of mentoring by female senior surgeons [16,17], as there are not many of them, with which we strongly agree. Mentoring could help encourage women to pursue a career as an orthopaedic physician. However, both men and women could take on the role of a mentor [18,19]. In addition, increasing the number of role models for women as orthopaedic surgeons, who can actively engage in academic activities as supervisors and managers through roles requested by meeting organizers, could serve as a benchmark for women as medical students and residents and would motivate young women (orthopaedic surgeons) to become more active in academic activities. Moreover, promoting the support and development of women with such ideas could increase the number of women as presenters in the future.

Female doctors in roles requested by the meeting organizers

The number of female invited speakers and seminar lecturers did not increase over time. However, the number of women as abstract reviewers, general abstract oral chairpersons, symposium chairpersons, and symposium lecturers did increase. Nevertheless, the number of women as symposium chairpersons and symposiasts depended on the availability of symposiums organized by the Gender Equality Committee and the Workplace Reform Committee. The percentage of women in these roles generally did not reach the percentage of women members of the JOA. There are two possible explanations for these results. First, since invited speakers and seminar lecturers at academic conferences are selected by those who have made academic achievements in their field, the proportion of them would decrease if women (physicians) present less at conferences. Second, relative underrepresentation of women in more important roles may exist, which may reflect an implicit gender bias on the part of the organizers of the JOA annual meetings. Such implicit negative gender biases have been shown to affect career opportunities and choices among women physicians negatively [15,16]. To improve gendered career paths and decrease implicit gender bias, meeting organizers could do better in providing information on bias awareness and raising awareness of this issue. On the other hand, the increasing trend for female symposium chairpersons and symposium speakers was in sync with the presence or absence of symposiums organized by the Gender Equality Committee or the Workplace Reform Committee, indicating the importance of the activities of the Gender Equality Committee and the Workplace Reform Committee in increasing the ratio of women in key roles.

Positive actions refer to actions taken to ensure that members of groups who have traditionally been treated unfairly for race, gender, or other reasons receive education, employment, or other benefits. Positive actions can involve a multiplicity of methods that may be useful for JOA gender equality. The most common methods include setting specific benchmarks for recruiting women to key positions in academic societies. These can be subdivided as follows: (1) A quota system, a method in which a certain number or percentage of women are assigned to key positions. (2) A goal and timetable system, a method in which goals are set to be achieved within a particular timeframe, and efforts are made to achieve these. (3) A plus-factor system, a method in which one is given priority if abilities are equal. In particular, the lack of role models and mentors, and the negative stereotypes in orthopaedics in Japan could be improved by introducing a specific benchmark for recruiting women to the roles requested by the organizers of the JOA annual meeting, i.e., by implementing the so-called quota system.

By increasing the role of women at annual JOA meetings, women are likely to take on increased roles at both regional and JOA-related meetings. This will present an opportunity to provide role models and mentors for women as residents and medical students who attend these meetings, help shape positive stereotypes of gender diversity in the JOA, and improve the implicit gender diversity bias.

Characteristics by subspecialty

Knee, paediatrics, and spine were the top three subspecialties that most frequently involved women as physicians in this study. Among the JOA-affiliated societies, paediatrics has the highest proportion of women (15%), while lower-extremity joint surgery and spine surgery have lower proportions of women physicians [4]. Thus, the identification of paediatrics as a top subspeciality is plausible based on the ratio of women as physicians in the orthopaedics department. The fact that knee and spine ranked high despite being subspecialties with a low proportion of women may reflect either the efforts of women (physicians) in these areas or the leadership of their supervisors at their institutions. To enhance the academic engagement of women as orthopaedic surgeons, we require support and collaboration from professionals across all specialties, not only in knee, pediatric, and spine surgery.

Strengths and limitations

Our study has several strengths. First, this study is the first to examine the engagement of women as orthopaedic surgeons in the JOA. Second, we found positive trends such as the gradual increase in the ratio of women among JOA members and their involvement in academic activities. Third, the study's longitudinal analysis covering a span of 11 years (2012-2022) provided a comprehensive view of trends over time.

However, it has several limitations. First, our data were associated with a risk of gender misclassification. To reduce the risk and increase accuracy, manual searches were conducted with multiple reviewers using search engines (e.g., Google) in cases where it was difficult to determine from the name. However, with the very low percentage of women as chairpersons and speakers in our study, the misclassification of gender might have affected the percentage of women as physicians. Second, to determine which subspecialties had more women as physicians, we surveyed the subspecialties of the first speakers of the oral and poster presentations. However, this might not have been consistent with the speaker's intentions, since any overlap of subspecialties in the abstracts was determined based on which session they were included in. Additionally, it was possible that the areas of subspecialties presented might not be consistent with the presenter's subspecialties. Third, the top three subspecialties with the most women as presenters at the JOA annual meetings were indicated to be knee, paediatrics, and spine. However, to begin with, knee and spine had a higher total number of presentations in their categories than did the other subspecialties, which does not mean that the percentage of women as physicians in those subspecialties is higher. Fourth, we used data from JOA and publicly accessible abstracts; possible variations in data quality or completeness might have occurred. Fifth, the study focused on a specific medical association in Japan. Therefore, whether the results can be extended to other orthopaedic associations or medical specialties remains unclear. Sixth, we could not investigate other departments on this topic. Further investigations on this topic without these limitations are needed to draw definitive conclusions.

## Conclusions

Although the proportion of women as members of the JOA has been increasing gradually and more women have been involved in JOA annual meetings, the proportion of women as presenters, invited speakers, symposiasts, and chairpersons of oral and poster presentations has generally been lower compared with the proportion of women as JOA members. More active education for women (physicians) in educational institutions and the creation of benchmarks for the selection of women as physicians for more important roles (chairpersons, educational speakers, and symposiasts) in the organization of JOA annual meetings are needed. In other words, raising awareness, including positive action, is required for JOA members. Improving gender diversity in JOA academia would increase the number of JOA-associated women (physicians) and their research capacity, which would be of great benefit not only to JOA but also to patients. This requires the efforts of all JOA members.
